# Case Report: Salvation of a congested SCIP flap with a modified “chemical leech” technique

**DOI:** 10.3389/fsurg.2024.1436599

**Published:** 2024-11-20

**Authors:** Yong Zhao, Xianquan Yang, Shaobo Zhu, Aixi Yu

**Affiliations:** ^1^ Department of Orthopedic Trauma and Microsurgery, Zhongnan Hospital of Wuhan University, Wuhan, Hubei, China; ^2^ Department of Orthopedics, Gucheng County Hospital, Xiangyang, Hubei, China

**Keywords:** chemical leech, flap, venous congestion, blood drainage, venous crisis

## Abstract

The superficial circumflex iliac artery perforator (SCIP) flap is a widely accepted workhorse flap for covering defects. Although the success rate of SCIP flaps is currently high, flap failure occurs occasionally due to venous congestion. Venous re-anastomosis is the ideal rescue method but is sometimes limited by poor venule condition. The “chemical leech” technique could relieve venous congestion without venous re-anastomosis. However, owing to insufficient offloading, this technique is less effective in free flaps than in fasciocutaneous flaps, especially large-volume flaps. In this case report, we modified the “chemical leech” technique by adding a venous catheter. Congested blood was drained in a 2-way manner, both through a venous catheter and the skin incisions. On the first day, congested blood was mainly drained through the catheter. Intermittent heparin irrigation was required to maintain the blood flow. On days 2 and 3, as the microcirculation improved, the flow regulator was turned down to reduce blood loss. Blood loss through the catheter decreased dramatically from day 4 onward. This was probably due to thrombosis in and around the catheter. Another pathway through the skin still worked until the establishment of microcirculation, which occurred on day 8. Compared to previous “chemical leech” therapy, the modified “chemical leech” therapy was more reliable and could help drain the congested blood on venule level in addition to capillary level, making the blood drainage more efficient.

## 1 Introduction

The superficial circumflex iliac artery perforator (SCIP) flap is an evolved form of superficial circumflex iliac artery (SCIA) flap that was first used as a free flap in 1973 by Dr. Talor (1). In 2004, Dr. Koshima revisited the SCIA flap based on the perforator rather than the main trunk of the SCIA, called the superficial circumflex iliac artery perforator (SCIP) flap (2). Since then, SCIP flap has been widely accepted as a workhorse flap due to the easy concealment of the donor site, minimal harvesting morbidity, and pliable nature to cover a defect. Although the SCIP flap is now performed with a very high success rate, flap failures do happen (3). Those failures are mainly caused by venous congestion. This may later cause compromised microcirculation featured with microcirculatory thrombosis, trapping of platelets, and stasis (4). Once congestion is identified, emergency intervention is required to establish venous outflow (5–7).

Venous re-anastomosis is an ideal procedure for free-flap congestion (8). However, in some cases, vascular re-anastomosis cannot be performed because of poor vascular condition (9). Additionally, similar to our case, due to severely damaged microcirculation, anastomose venules are pointless because there is little to no blood outflow. Even though there was blood flow, the volume was relatively small compared to normal venous return, and re-anastomosing the vessel posed a risk of re-thrombosis (10). Therefore, for flap venous crises where venous re-anastomosis is not suitable, alternative simpler and safer methods may be needed to improve flap perfusion. First reported by Barnett et al. in 1989, the “chemical leech” technique is considered helpful for relieving venous congestion. In that study, local subcutaneous heparin was injected subcutaneously, which alone led to satisfactory results in the digital replantation scenario (11). Following this, scarification or needle puncture after heparin injection (12). However, because of insufficient offloading, this technique is less effective in free flaps than in fasciocutaneous flaps, especially in large-volume flaps (13). The venous catheterization technique, in which a venous catheter is inserted to one of the donor veins of the ﬂap to release venous congestion, has been also developed as an option for venous offloading (7).

In this paper, we propose a modified “chemical leech” technique by combined the “chemical leech” technique and the venous catheterization technique. This study is also the first to focus on a solution to SCIP flap congestion. Having experienced desperation after witnessing the purple, cold, and swelling flap, and then the excitement after watching the congestion gradually fading away, we would like to share the method we use and the experience we learned. Hopefully, someone who is unfortunately trapped in a similar situation may benefit from this.

## 2 Patients and methods

**Figure 1:**
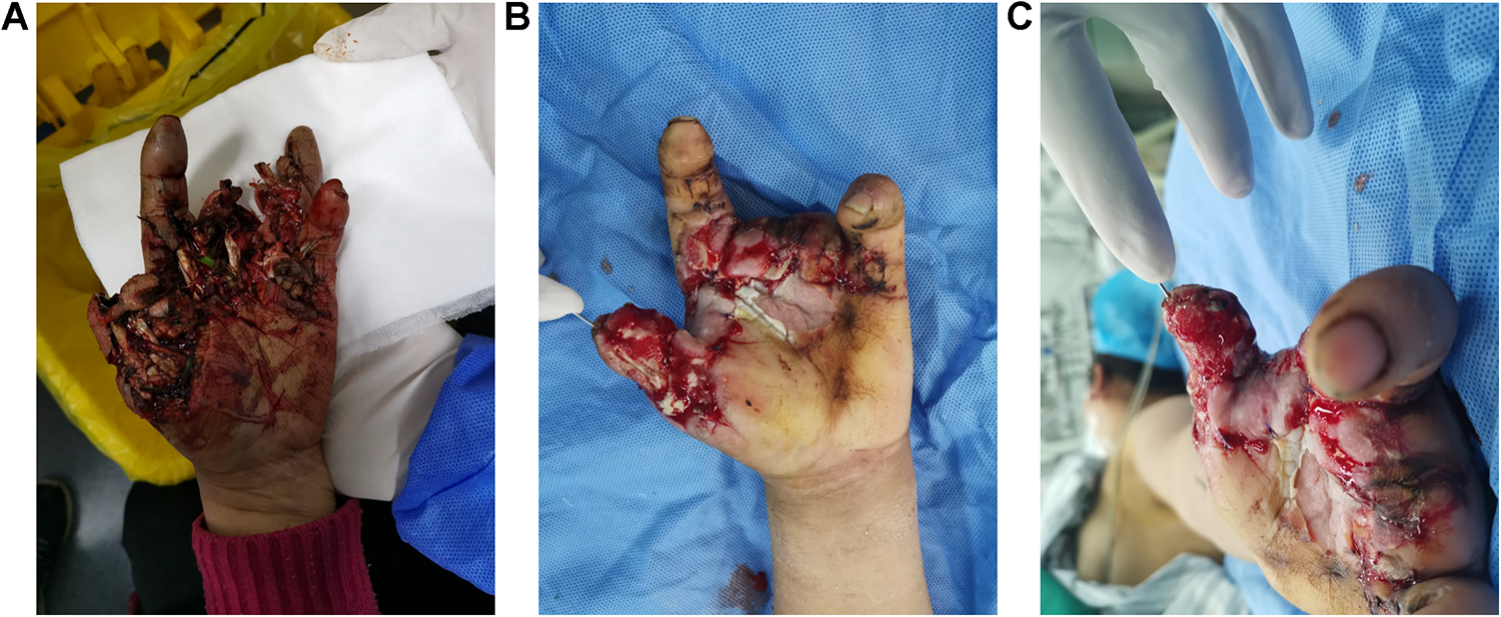
Images of the patient's hands. **(A)** Before debridement. **(B,C)** A week after debridement.

The patient was a 53-year-old female. When preparing food for farm animals, she accidently put her hand in the inlet of the grass grinder and was hurt by the blunt blades. As shown in Figure 1A, the entire hand was contaminated with grass and dirt. The soft tissue of the patient's thumb was managed by using a dislocated interphalangeal joint. Blood supply to her index finger was compromised by a broken ulnar digital artery and soft tissue damage. Anastomosis of the ulnar digital artery of the index finger was performed by using a venous graft harvested from the forearm. The middle and ring fingers were smashed into pieces and were unqualified for replantation. The dorsal and proximal parts of the skin of the middle and ring fingers remained viable and were used as local skin flaps to cover the palm defect. After thorough irrigation and debridement, the dislocated interphalangeal joint of the thumb was reduced and fixed using K-wire. Negative pressure wound therapy (NPWT) was applied to cover and irrigate the wounds. One week after debridement, the wound was relatively clean, and soft-tissue defects were observed on the palmar side of the metacarpophalangeal joint (Figures 1B,C).

### 2.1 SCIP flap transfer

**Figure 2:**
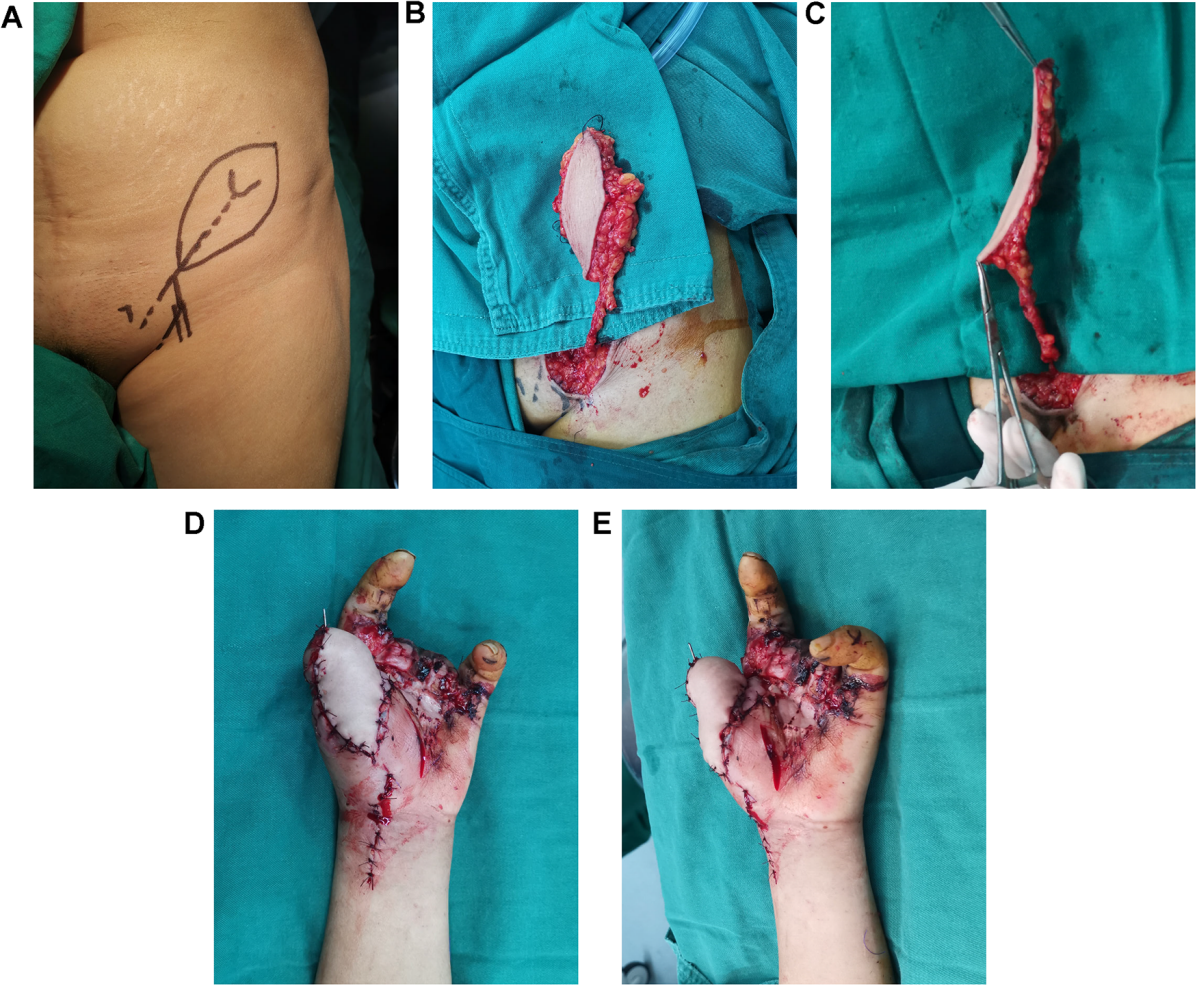
SCIP flap design, harvest and transplantation. **(A)** Flap design. **(B)** Thick flap with large fat lobule. **(C)** Thinned flap after removing the large fat lobule. **(D,E)** Transferred flap.

The course of the SCIP was traced using bidirectional Doppler (ES 100V3, Hadeco, Japan). A medial perforating branch was used. A z-shaped incision (Figure 2A) was made on top of the medial branch. Once the medial branch was identified, the flap was raised above the deep fascia. Large deep fat lobules were observed under a microscope, whereas the superficial fat lobules were preserved (Figures 2B,C). When the flap was transferred to the thumb, the arteriole was anastomosed to the superficial branch of the radial artery and the accompanying venule to the superficial vein with a similar caliber, both in an end-to-end manner.

### 2.2 Venous congestion identified and re-exploration surgery

Flap surgery was completed at 2 p.m. on 03/03/22 (day 0) and checked every hour. The circulation was confirmed to be fine at approximately 10 pm. However, on the next day (day 1) around 8 a.m., the flap turned purple, cold, and swollen. Thus, we know that severe congestion occurred. Emergency re-exploration surgery was immediately performed. A thrombus was identified at the site of the venous anastomosis. No blood outflow was observed after the thrombus removal. We then used heparin to irrigate the venule for approximately half an hour, as tiny clots were flushed out, and finally, some venous blood oozed out of the venule. When irrigation was stopped for 3–5 min, the outflow gradually stopped. The situation did not improve even when irrigation was continued for another hour. The re-anastomosis appeared to be pointless, since that the venous volume was relatively small compared to normal venous return, and re-anastomosing the vessel posed a risk of re-thrombosis. Additionally, at this stage, the vessels were fragile and edematous, which might necessitate venous grafting, thereby increasing the likelihood of failure. Thus, we decided to use the modified “chemical leech” therapy. The detailed methodology of the modified “chemical leech” technique is presented below.

### 2.3 Modified “chemical leech” therapy

**Figure 3:**
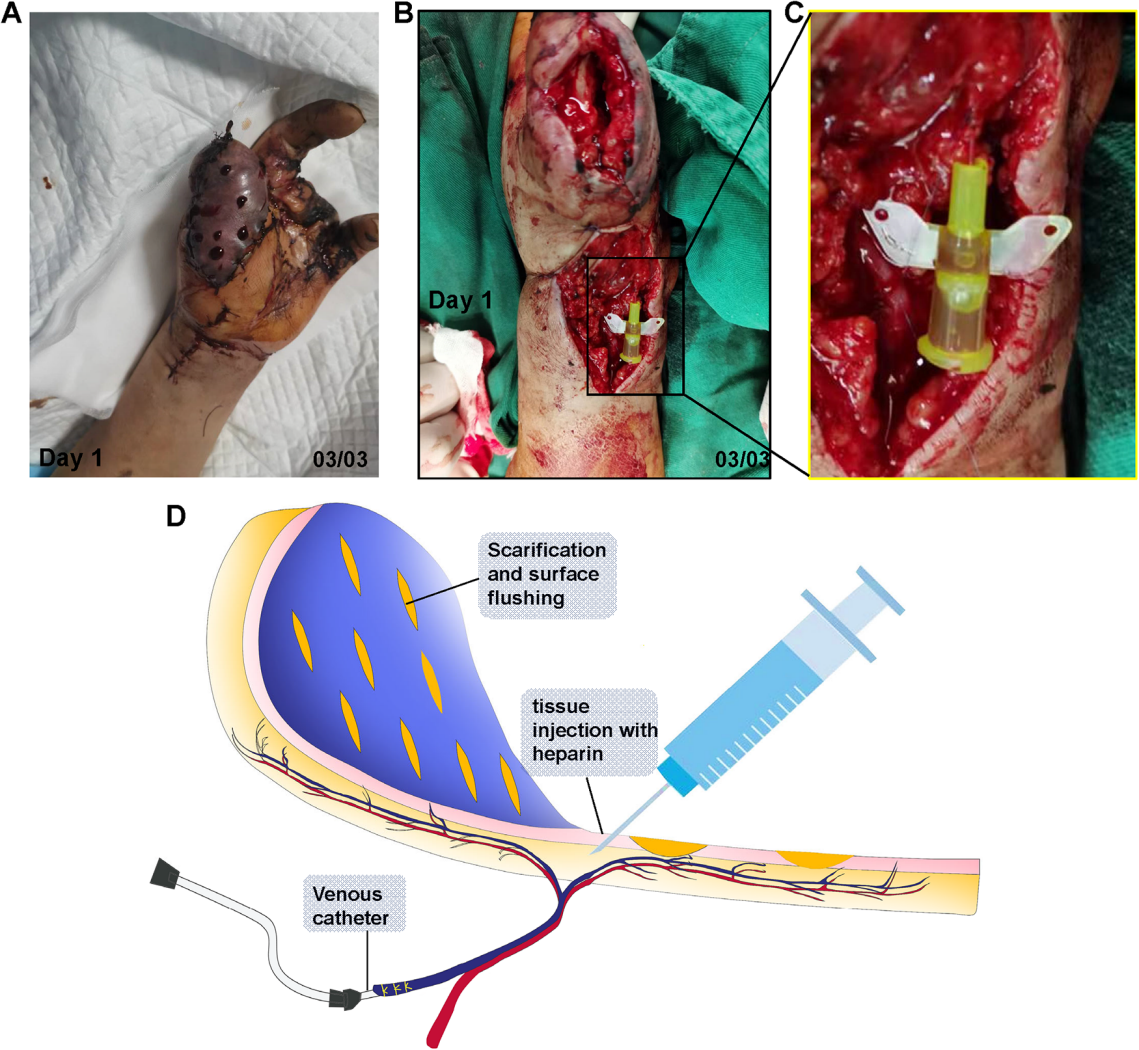
Salvation of SCIP flap with the modified chemical leech therapy. **(A)** Scarification and subcutaneous heparin injection on the congested flap. **(B)** Venule catheter placement. **(C)** Magnification of the catheter in **(B–D)** illustration of modified “chemical leech therapy”, which conclude venous catheterization, scarification and tissue injection.

As shown in Figure 3, the therapy consisted of three parts: (1) Subcutaneous heparin injection. The injection protocol was based on both the evidence established by Barnett et al. (12) and the actual clinical situation. Protocol: 1st day: 500 IU (Fluxum, 3,200 IU, Italy) IH, Q8 h; 2nd day: 250 IU IH, Q12 h; 3rd day: No injection administered because of apparent blood loss, 4th-7th:100IU IH, Q12 h; (2) Scarification. A nearly parallel (to the axis of the flap) cut was made using a #11 blade throughout the full thick skin, and the length and density of the cuts varied and were decided by the surgeon based on the area and condition of the flap; and (3) venous catheter placement. An appropriate catheter should be selected based on the size of the venule. In this case, a 26 G indwelling needle was placed inside the venule, 7-0 sutures were used to tie the venule and the needle together. After the successful venous catheterization, no significant blood outflow was observed. Therefore, we continued to flush with heparinized saline, after which some venous blood oozed out of the catheter. The specific irrigation method was detailed below.

### 2.4 Irrigation process of the catheter

On day 1, immediately after re-exploration, irrigation was performed every 5 min to maintain venous blood flow. The amount of irrigation required depended on the goal of keeping the catheter unobstructed. This task was both time consuming and tedious. After approximately 3 h, we found that a 10 min interval was sufficient to keep the catheter unobstructed. Then, 30 min, and eventually 1 h at the end of day 1. On day 2, a 2-h interval for irrigation during the daytime was adopted. No irrigation was performed during the night because there was a continuous yet small amount of blood oozing out, even without irrigation. On day 3, the needle was sealed every hour for 30 min to prevent excessive blood loss. On day 4, the amount of outflowing blood decreased dramatically and no sealing was performed. In contrast, hourly interval irrigation was used. On day 5, the irrigation was stopped because there was no outflow of blood from the needle.

### 2.5 Blood outflow profile

The blood outflow profiles from both the catheter and skin incisions were monitored and recorded. The gross blood loss was calculated using the following equation: Blood loss volume≈Ww-Wd-Vi (Ww: weight of wet dressing; Wd: weight of dry dressing; Vi: Irrigation Volume).

## 3 Results

### 3.1 Blood outflow profile from the catheter

On day 1, moderate venous blood (approximately 50 CC) was collected from the catheter. On day 2, it was estimated that approximately 300 CC of venous blood had drained from the catheter. On day 3, blood loss was approximately 500 cc even with intermittent catheter sealing. On day 4, the amount of outflowing blood decreased dramatically and only approximately 50 CC of blood were drained. On day 5, the needle dried out and no blood outflow was observed, even with irrigation.

### 3.2 Blood outflow profile from the skin incisions

On day 1, immediately after scarification, dark red congested blood was removed. After a few minutes of heparin irrigation, fresh arterial blood was removed. On day 2, the amount of congested blood decreased sharply compared to that on day 1; the oozing out of the blood was mainly arterial blood with a bright red color. On days 3 and 4, instead of blood, the outflowing fluid was mainly transparent exudate. On day 5, only 1 of the 16 cuts remained functional, whereas the rest were closed. On day 8, all the cuts were closed.

### 3.3 Changes of the flap over time

**Figure 4:**
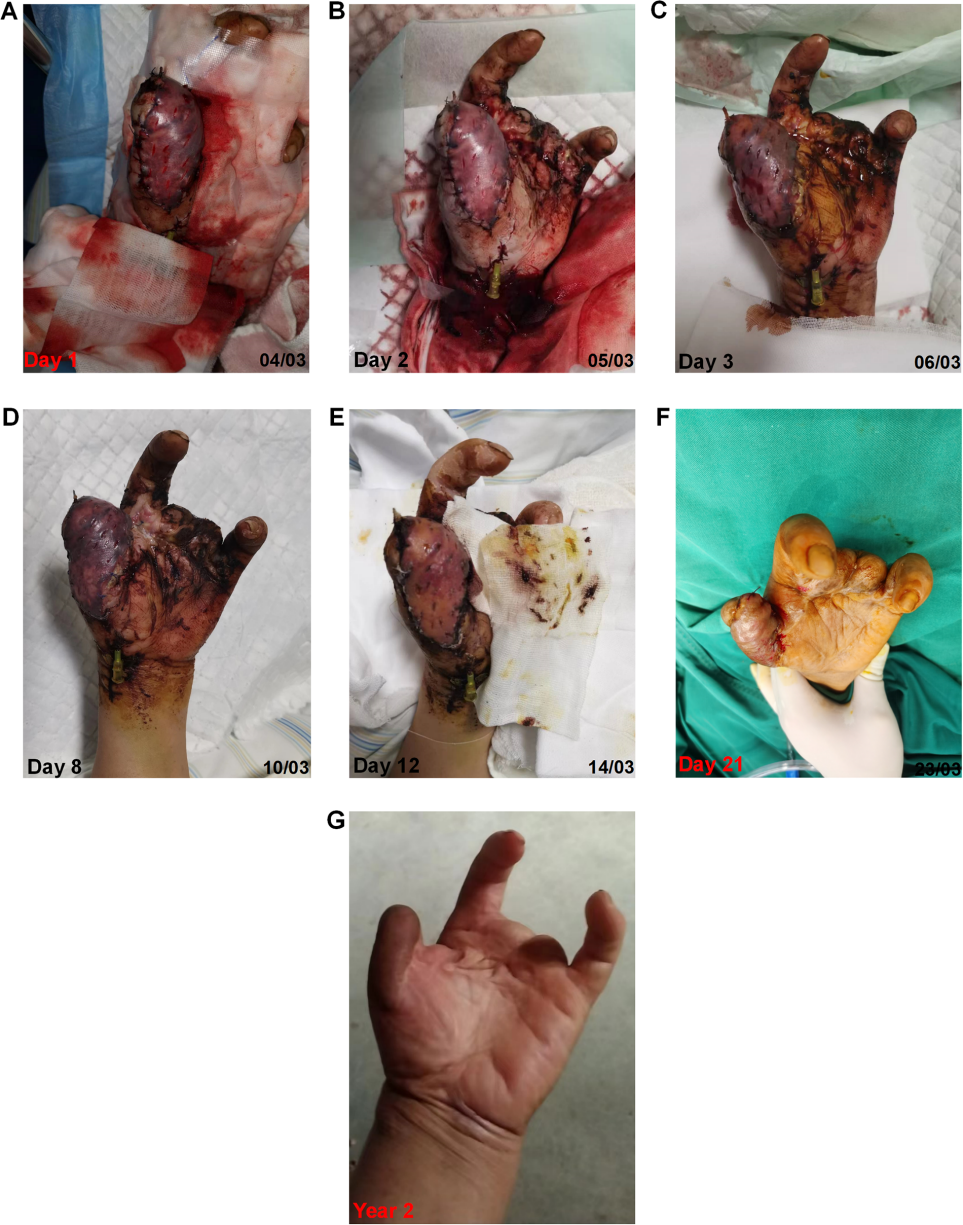
Changes of the congested flap over time under the modified “chemical leech therapy”.

As shown in Figure 4, on day 1, the flap was dark purple in color, with a low temperature and high tension. The capillary refill test was not applicable, because the flap did not turn white upon local compression. On day 2, approximately 2/3 of the flap turned dark red, and the subcutaneous tissues (seen from the cuts) exhibited a slightly brighter red color. On day 3, the color of the flap became pink-like and apparent edema was observed. On day 8, one could clearly see the shrinking of the flap, and the congestion of the flap changed to dark cloud fading. On day 12, most flaps had a healthy pink color. On day 21, the flap completely survived. Two years postoperatively, the flap appearance was good, and the patient's thumb function was excellent (Supplementary video material).

## 4 Discussion

Similar to other free flaps, the SCIP flap is susceptible to venous crisis. Likewise, venous congestion occurs much more frequently than arterial crisis in SCIP flaps, mostly because of the lower pressure of the venule compared to the arteriole (10, 14). In particular, venous crisis of the SCIP flap is a little trickier than that of other flaps because the venule is small (sometimes less than 0.5 mm) and delicate (15, 16). Various methods are available for venous congestion, as mentioned in the Introduction (7).

In this case, an attempt to perform re-anastomosis failed due to venous outflow merely after removing the thrombus. Thrombolytic therapy and subsequent vascular re-anastomosis were considered. However, in this patient, the small calibre of the SCIP and the awkward position of the anastomotic site forced us to think about the other way. Medical leech therapy is considered the gold standard among the nonsurgical methods (17, 18). Unfortunately, our unit, like many other units around the world, does not have medical leeches.

As shown in Figure 3, we proposed a “modified chemical leech technique” to treat venous congestion. In this technique, venous catheterization was added to the previous “chemical-leech” therapy. Thus, congested blood was drained in a 2-way manner, both through the venous catheter and skin incisions. In terms of the mechanism, the modified “chemical leech” therapy helped drain the congested blood on venule level in addition to capillary level compared to previous “chemical leech” therapy. Therefore, blood drainage was more efficient. On the first day, congested blood was drained using a catheter. Intermittent heparin irrigation is required to maintain blood flow. On days 2 and 3, as microcirculation improved, the flow regulator was turned down to reduce blood loss. Blood loss through the catheter decreased dramatically from day 4 onward. This was probably due to thrombosis in and around the catheter. Another pathway through the skin still works until the establishment of microcirculation, which occurs on day 6.

Compared to previous “chemical leech” therapies, this modified technique has 3 advantages: (1) More efficient in relieving congestion owing to 2-way drainage; (2) More reliable, when one of the two methods fails, the other method still works; and (3) With a flow regulator, the venous outflow is relatively controllable. However, there are problems associated with this technique, such as blood loss and catheter blockage. Therefore, blood loss must be carefully monitored when using this technique. For unstoppable bleeding from the skin, gentle pressure with gauze can be applied until the bleeding ceases. The flow was reduced for massive bleeding through the catheter. Blood transfusions were required when necessary.

Since we had only one case, more cases need to be recruited to test the safety and efficacy of this technique. In future, this technique can be used not only for SCIP flaps but also for other free flaps or fasciocutaneous flaps. With proper monitoring and handling, we believe “modified chemical leech technique” can be a problem solver for certain kind of venous congestion.

## 5 Conclusion

The modified “chemical leech” therapy was reliable and could help drain the congested blood on venule level in addition to capillary level, making the blood drainage more efficient than the previous “chemical leech” therapy.

## Data Availability

The original contributions presented in the study are included in the article/Supplementary Material, further inquiries can be directed to the corresponding authors.
